# Magnetic Resonance Imaging-Based Connectomics in First-Episode Schizophrenia: From Preclinical Study to Clinical Translation

**DOI:** 10.3389/fpsyt.2020.565056

**Published:** 2020-09-11

**Authors:** Jin-Bo Jiang, Yang Cao, Ning-Yu An, Qun Yang, Long-Biao Cui

**Affiliations:** ^1^ Department of Clinical Psychology, School of Medical Psychology, Fourth Military Medical University, Xi’an, China; ^2^ Department of Radiology, The Second Medical Center, Chinese PLA General Hospital, Beijing, China

**Keywords:** schizophrenia, connectome, MRI, early identification, diagnosis

## Introduction

Schizophrenia is a debilitating mental disorder (1). Schizophrenia affects the emotional, behavioral, and cognitive aspects of patients, causing a serious burden on the society, so early identification and intervention in schizophrenia is urgent. To improve management of schizophrenia, a better understanding of its neural mechanism is required. Emerging preclinical studies aim to rely on magnetic resonance imaging (MRI) to look for underlying neural abnormalities in patients with schizophrenia, in order to provide potential imaging markers for the early identification and diagnosis of schizophrenia. The connectomics is the state-of-the-art technology. Schizophrenia is a disorder of connectome development (2) with a large consensus in connectomics literatures (3). On the microscale, connectome refers to a complete map of the wiring of an organism’s nervous system (4). On the macroscale, the commonly used techniques to construct connectomics include electroencephalogram (5), positron emission tomography (6), and most commonly the MRI. During the past several years, it has been demonstrated that abnormal connectome organization exists throughout the whole course of schizophrenia, including the status at risk for the disorder (7–12). With converging evidence of analysis suggesting that disrupted connectome may be a core phenotype of schizophrenia (13), a review on this topic seems to be critical and urgent.

## Magnetic Resonance Imaging-Based Connectomics in First-Episode Schizophrenia

### Genetic Influence on the Early Stages of Schizophrenia

When talking about the early stage of schizophrenia, genetic factors play important roles (14). Schizophrenia is a complex and multi-gene inherited mental disorder, and this leads to disorder of the connectome in the brain, so the emergence of connectomics provides indirect evidence for the study of genetic factors in schizophrenia. Genetic studies have found that the closer relatives are, the greater the risk of developing the disorder. For example, if a person developed schizophrenia, his first-degree relatives — his parents, children, and siblings have 6 to 10 times higher risk of being affected than general population (15). Genetic predisposition to schizophrenia is manifested both in brain structure and function. Many studies were conducted in individuals at high risk of schizophrenia, and it has been revealed that the middle temporal, frontal, cingulate, and occipital lobes were thinner in the first-degree relatives of schizophrenia patients than healthy controls (16–18). Genetic predisposition is also demonstrated in brain physiology, including increased blood flow in areas of the default mode network (DMN) in patients’ relatives comparing to healthy controls (19). Blood flow networks in the brains of patients were impaired, as was the connection between the anterior cingulate cortex and the hippocampus (19). When the brain is at resting state, there are still patterns of co-activation that recapitulate brain network co-activated during task performances (20). DMN is a set of brain regions that show decreased activity during tasks but increased activity at rest. It comprises the posterior cingulate cortex, precuneus, inferior parietal cortex, medial prefrontal cortex, and medial temporal lobe (21). More results have been accumulated in the analysis of brain activity in patients with schizophrenia using DMN. Mallikarjun et al. found that the resting seed-based functional connectivity between parts of the salience network (SN) and the DMN and between the SN and the cerebellum increased in first-episode psychosis patients, while the resting functional connectivity between claustrum and insular lobe decreased (22).

By combining genetic and multiparadigm MRI data of 623 healthy Caucasian adults drawn from the Human Connectome Project, Cao et al. found that higher schizophrenia polygenic risk scores were significantly correlated with lower functional connectivity in a large-scale brain network primarily encompassing the visual system, default-mode system, and frontoparietal system (23). Disrupted integration of sensori-cognitive information may be a hallmark of genetic effects on the brain that contributes to the pathogenesis of schizophrenia (24).

Schizophrenia is a complex psychiatric disorder. The genetic predisposition of schizophrenia may impose an influence on the development of brain connectivity. In this case, connectome provides a non-invasive method of examination and analysis. It provides a solution for assessing brain function and mental health.

### Application of Connectome in Diagnosis of Schizophrenia

The human brain is a complex network of interconnected brain regions, and connectomics provides a powerful way to understand the neuropathology of disease by mapping the brain’s neural connections with high precision and resolution (25, 26). In a recent study, Cui et al. recruited 42 first-episode, medication-naïve schizophrenia patients and 48 healthy controls (HCs) in the principal data set (27). Each patient was assessed at the time of scanning by using the Positive and Negative Syndrome Scale. As well, 39 first-episode patients (10 untreated patients) and 66 HCs were enrolled as the replication dataset. By using diffusion-weighted imaging (DWI) and resting-state functional magnetic resonance imaging (fMRI), they identified rich club regions in healthy controls, including bilateral superior frontal gyri, superior parietal lobules, insula, and left precuneus. Connections were classified into three categories: rich club, feeder, and local connections. By comparing rich club for first-episode medication naïve patients with schizophrenia and HCs, they found that rich club organization was significantly impaired in patients compared to HCs (27).

These results have important clinical implications for the early diagnosis of schizophrenia. A previous study confirmed that the diagnostic accuracy of screening brain features derived from resting-state fMRI to identify patients with schizophrenia is 87% (28). Since the subjects included in this study were first-episode patients, the effect of drug use on the brain connections of patients was effectively avoided. Finally, the result is comparable to previous studies, suggesting impairment of rich-club organization in schizophrenia is consistent across populations/ethnic groups (10–12).

The measures of connectomics can not only identify schizophrenia (29), but also provide clue for the clinical classification of schizophrenia. It is well known that schizophrenia is a group of clinical syndromes with high clinical heterogeneity (30). One of the hallmark symptoms of schizophrenia is auditory verbal hallucination (AVHs). Using support vector machine (SVM) Huang et al. 's research results suggest that functional connectivity can identify schizophrenia patients with AVHs, with an accuracy of 75.6% (31). The findings of connectomics contribute to this subtype of schizophrenia.

Additionally, it should be emphasized that connectome is a potential guide to treatment and prognosis of schizophrenia. Connectomics provides a reference for evaluating the efficacy of antipsychotics in schizophrenia. By analyzing MRI data of 22 patients and 20 healthy controls, Cadena et al. found that greater functional connectivity between anterior cingulate cortex and bilateral putamen at baseline predicted subsequent better treatment response (32). To investigate whether resting-state functional connectivity is associated with long-term clinical outcomes of patients with schizophrenia, Lee et al. found that poorer clinical outcomes in patients with schizophrenia were associated with decreased DMN connectivity (33). In particular, the decreased functional connectivity might be related to the severity of positive and mood symptoms rather than negative symptoms.

The early stage of schizophrenia is called prodromal stage. Prodrome refers to the onset of the general symptoms of the disease, to the typical symptoms of the disease before the onset (34). It can also be said that the period before the onset of pre-psychiatric psychosis is a deviation from a person’s previous experience and behaviour (35). In the prodromal stage, several typical symptoms are changes in mood, cognitive function, perception, behavior, and physical symptoms. When these symptoms occur, clinicians are unable to diagnose schizophrenia according to the diagnostic criteria (e.g., DSM-5), because the symptoms at this time may not meet the diagnostic criteria for symptoms, functional criteria, disease course criteria, and exclusion criteria. Early diagnosis is important for the treatment of schizophrenia. It has been shown that patients with longer period without effective treatment will have worse prognosis comparing to patients treated early (34, 36). The emergence of connectomics gives a solution to this problem to some extent. In the first place, we briefly view the connectomics in schizophrenia through the prism of translational medicine. Dysconnection hypothesis has been presented due to the development of neuroimaging (2, 37, 38), in particular MRI. The so-called “connectome” was introduced in 2005 for comprehensively describing the human brain network of neural elements and connections (39). Connectomics provides a powerful approach for understanding cognition (40) and neuropathology (4). Schizophrenia has been the main focus of connectomics research and was conceptualized as a brain network disorder. In recent years, several excellent reviews have summarized the history of connectomics and the findings of a rising number of studies of brain connectivity in schizophrenia [for review, see (41–43)]. Future studies might be helpful allowing objectively and precisely predicting, diagnosing, and monitoring schizophrenia. Abnormal cortical-striatal-cerebellar network connections has been found in schizophrenia (44). Consensus neuroimaging findings, such as a decreased rich club connectivity in patients, may help clinician to consider to diagnose schizophrenia in the future (27).

### Early Diagnosis of Schizophrenia

Neuroimaging has shed some light on the neurological changes that occur in the early stage of schizophrenia. We can take advantage of genetic characteristics and these changes to make an identification of schizophrenia at early course, which is the prodromal stage. As mentioned earlier, prodrome is important for the diagnosis and treatment of schizophrenia (34). Therefore, several tools were created to detect the prodromal stage of psychosis (45). Intervention in the prodrome stage has been reported to improve cure rates and prognosis (46). Therefore, it is very necessary to identify the individuals in the prodromal stage of schizophrenia.

**Figure 1:**
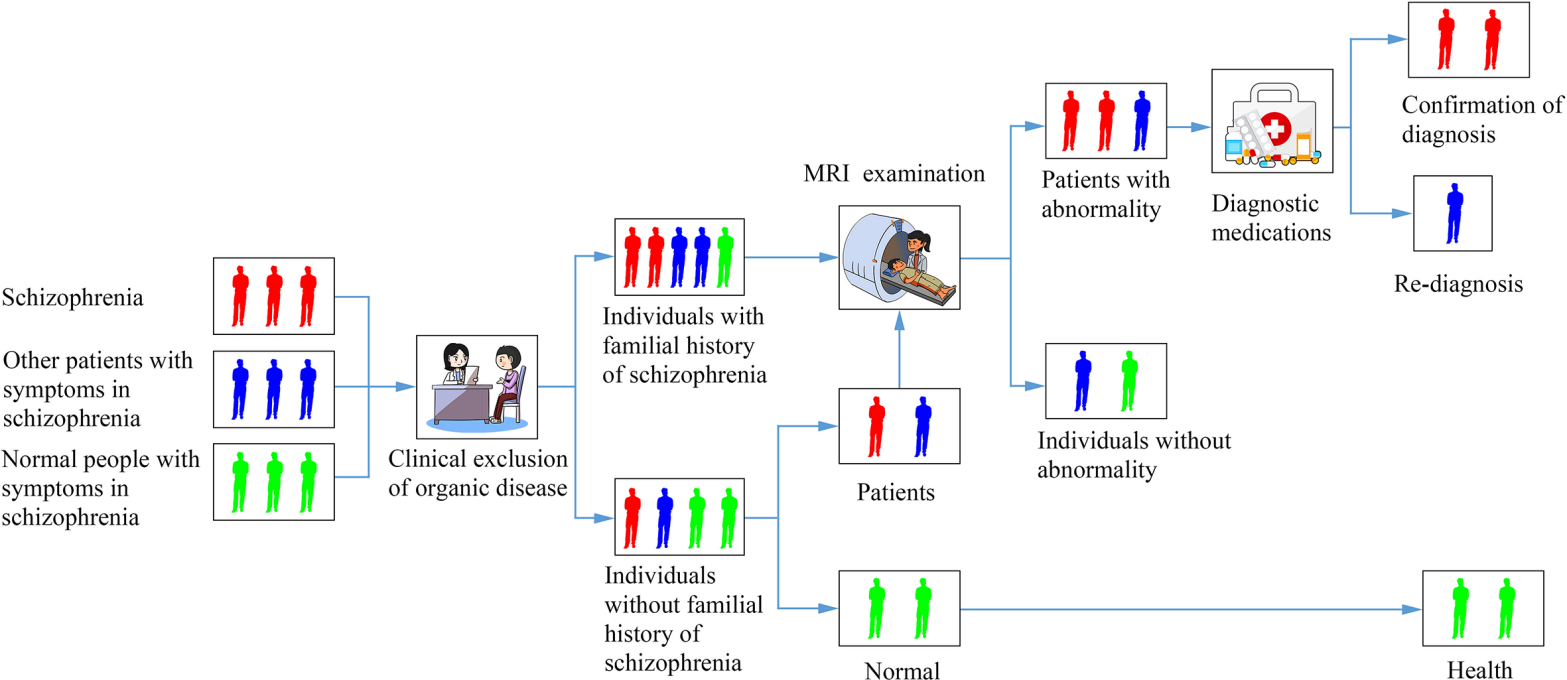
A diagram of a flowchart for identifying a patient with schizophrenia based on connectomics.

When a patient comes in with typical prodromal symptoms, such as changes in mood, cognitive function, perception, behavior, and the appearance of various somatic symptoms, the physician should first rule out somatic and neurological disorders which may lead to these symptoms. Clinical physicians ask the patient if there is a clear trigger or family history. If not, physicians observe them and pay attention to follow-up. If there is a clear trigger, the possibility of schizophrenia is highly suspected and patients should be referred for laboratory inspection, such as MRI. When the connectome results show normal, the diagnosis of schizophrenia could be initially excluded and follow-up observation could be conducted. If the imaging results show the results of previous study, such as the defect of thalamic-auditory cortex-hippocampal connection (47), abnormal cortical-striatal-cerebellar network connections (44), the initial diagnosis of schizophrenia can be made. Diagnostic medications can be given at this point, and if they are effective for symptom relief, then schizophrenia can be diagnosed. If treatment does not work, the diagnosis should be re-considered. At the same time, the diagnostic process should be pushed back to the beginning for re-diagnosis (**Figure 1**). The validity of this prodrome diagnosis process of schizophrenia remains to be verified by big data.

## Discussion

One of the major difficulties in current connectome studies is that there are not enough evidence to evaluate the sensitivity and specificity of the diagnostic method. Meanwhile, most studies were based on a small sample and may have insufficient statistical power to detect disease-related changes (28). Further, studies focusing on group-comparisons could not make inference to individual cases. In the following research, data collection should be continued first, the sample size should be expanded, and more patient data should be collected for big data analysis, such as ENIGMA Schizophrenia (48, 49), so as to summarize a set of criteria that meet the diagnosis of most schizophrenia patients. At the same time, the existence of individual differences should also be considered. If there are special cases, that is, patients show unusual abnormal imaging manifestations, they should not be discarded, but should be included in the diagnostic criteria after careful analysis, so as to better improve the sensitivity of the diagnostic criteria.

We need to thoroughly study the mechanism of schizophrenia and clarify its occurrence and developmental course. Through this discovery, we can provide more objective imaging evidence for the prodrome of schizophrenia diagnosis. The current hypothesis for positive symptoms of schizophrenia, auditory hallucinations, involves many disciplines such as neuroimaging, neurobiochemistry, and neuropathology (50). Therefore, future research could benefit from research findings from different levels of studies. We should improve the objective laboratory examination for the prodrome of schizophrenia, such as establishing a complete set of scoring criteria based on neuroimaging, neurobiochemistry, and neuropathology, so as to effectively improve the detection probability of schizophrenia at early course, and thus truly benefit the patients with schizophrenia.

## Author Contributions

L-BC conceptualized the manuscript. J-BJ and L-BC wrote the first draft of the manuscript. All authors contributed to the article and approved the submitted version.

## Funding

This work was supported by the grant support of Fourth Military Medical University (2019CYJH), Project funded by China Postdoctoral Science Foundation (2019TQ0130), and National Natural Science Foundation of China (81801675).

## Conflict of Interest

The authors declare that the research was conducted in the absence of any commercial or financial relationships that could be construed as a potential conflict of interest.
